# FRET Visualization of Cyclic Stretch-Activated ERK *via* Calcium Channels Mechanosensation While Not Integrin β1 in Airway Smooth Muscle Cells

**DOI:** 10.3389/fcell.2022.847852

**Published:** 2022-05-19

**Authors:** Xin Fang, Kai Ni, Jia Guo, Yaqin Li, Ying Zhou, Hui Sheng, Bing Bu, Mingzhi Luo, Mingxing Ouyang, Linhong Deng

**Affiliations:** Institute of Biomedical Engineering and Health Sciences, School of Pharmacy & School of Medicine, Changzhou University, Changzhou, China

**Keywords:** cyclic stretch, mechanosensation, ERK, calcium channel, fluorescence resonance energy transfer

## Abstract

Mechanical stretch is one type of common physiological activities such as during heart beating, lung breathing, blood flow through the vessels, and physical exercise. The mechanical stimulations regulate cellular functions and maintain body homeostasis. It still remains to further characterize the mechanical-biomechanical coupling mechanism. Here we applied fluorescence resonance energy transfer (FRET) technology to visualize ERK activity in airway smooth muscle (ASM) cells under cyclic stretch stimulation in airway smooth muscle (ASM) cells, and studied the mechanosensing pathway. FRET measurements showed apparent ERK activation by mechanical stretch, which was abolished by ERK inhibitor PD98059 pretreatment. Inhibition of extracellular Ca^2+^ influx reduced ERK activation, and selective inhibition of inositol 1,4,5-trisphosphate receptor (IP_3_R) Ca^2+^ channel or SERCA Ca^2+^ pump on endoplasmic reticulum (ER) blocked the activation. Chemical inhibition of the L-type or store-operated Ca^2+^ channels on plasma membrane, or inhibition of integrin β1 with siRNA had little effect on ERK activation. Disruption of actin cytoskeleton but not microtubule one inhibited the stretch-induced ERK activation. Furthermore, the ER IP_3_R-dependent ERK activation was not dependent on phospholipase C-IP_3_ signal, indicating possibly more mechanical mechanism for IP_3_R activation. It is concluded from our study that the mechanical stretch activated intracellular ERK signal in ASM cells through membrane Ca^2+^ channels mechanosensation but not integrin β1, which was mediated by actin cytoskeleton.

## Introduction

Mechanical forces associated with cyclic stretch play important roles in the control of vascular functions and pulmonary circulation homeostasis, and stretch exercise in ordinary life shows benefits in improving physical and mental health (Geneen et al., 2017; Fang et al., 2019). In medical emergency, however, mechanical ventilation with repetitive cyclic stretch can result in inflammation and lung tissue injury (Slutsky and Ranieri, 2014; Horie et al., 2016). In the past decades, mechanical stretch in regulating physiological functions and cellular signaling has attracted wide research interests (Sadoshima and Izumo, 1993; Loperena et al., 2018; Chu et al., 2019; Ren et al., 2021). The mechanotransduction mechanism of mechanical stimulation to cellular biochemical signals has been a topic that is yet to be fully understood.

Physiologically, mechanical stimulations show fundamental roles in cell behaviors, tissue and organ developments, and disease-associated processes (Ingber, 2003; Mammoto et al., 2013; Qi et al., 2016). Several mechanisms have been discovered for the mechanical stimulations to biochemical signal transformations including cellular mechanosensitive components and extracellular matrix (Swaminathan and Gloerich, 2021). For instance, Polycystin-1, a large-size molecule with eleven transmembrane domains, has been shown to act as the mechanosensing component to modulate mechanical stretch-induced bone-cell differentiation (Dalagiorgou et al., 2013; Dalagiorgou et al., 2017). Membrane-localized ion channels TRPV4 and Piezo2 played central roles in calcium oscillation induced by physiological (8%) and injurious (18% of strain) levels of mechanical strains in chondrocytes, respectively (Du et al., 2020). Kim et al. investigated the mechanism of cellular Ca^2+^ signaling induced by pulling force using optical laser tweezers, and found that the endoplasmic reticulum (ER) Ca^2+^ release is mediated by both actin cytoskeleton and mechanosensitive Ca^2+^ channels on the plasma membrane (Kim et al., 2015).

Cellular biochemical activities are regulated by mechanical stretch at multiple levels. It has been observed for long that cells reorient themselves nearly perpendicular to the direction of cyclic stretch, and so does the actin cytoskeleton (Hayakawa et al., 2001; Wang et al., 2001; Jungbauer et al., 2008; Liu et al., 2008). Except for morphological changes, ATP release is visualized by induction of mechanical stretch in human airway smooth muscle (ASM) cells (Takahara et al., 2014), and the ATP release is regulated by Ca^2+^ signaling via caveolae-mechanosensitive pathway in alveolar epithelium (Diem et al., 2020). The level of total reactive oxygen species by mitochondrial and NADPH oxidases is increased under mechanical stretch in retinal pigment epithelial cells (Liang et al., 2019). Cyclic mechanical stretch also increases β_1D_-integrin protein level and activates the downstream signaling proteins focal adhesion kinase (FAK) and RhoA (Zhang et al., 2007). The abundance of CD40 in endothelial cells is upregulated through transforming growth factor β1 signaling when co-cultured with smooth muscle cells under cyclic stretch stimulation (Korff et al., 2007). A recent study identified mechanical stretch-mediated transcriptome profile changes during skin regeneration including nine robust hub genes and six transcriptional factors–mRNA regulatory network (Liu W. et al., 2021). Cyclic stretch also regulates extracellular secretions of vascular smooth muscle cells including microvesicles and growth factors, leading to functional modulations of surrounding cells and tissues (Wang et al., 2017; Liu J.-T. et al., 2021).

Mechanical stretch has been shown to stimulate the MAPK family ERK1/2 and c-Jun NH2-terminal kinase (JNK), depending on the stress fiber strain but not FAK in endothelial cells (Correa-Meyer et al., 2002; Hsu et al., 2010; Schmidt et al., 2016). Alexander et al. reported stretch-induced ERK1/2 activation mediated by phospholipase A_2_ (PLA_2_)-dependent release of arachidonic acid in renal epithelial cells (Alexander et al., 2004). Myosin II-regulated tension on the stress fibers shows positive correlation with ERK activation in fibroblasts (Hirata et al., 2015). Kim et al. reported that in mechanical stretch-induced loss of myelin proteins, the release of Ca^2+^ from endoplasmic reticulum (ER) resulted in ERK activation in oligodendrocytes (Kim et al., 2020). Although ERK activation by mechanical stretch has been well documented, it still remains to further characterize the mechanosensitive pathway for the mechanical-biomechanical coupling to activate ERK kinase.

Airway smooth muscle (ASM) cells, one major component of bronchial tissue underneath the epithelia, provide mechanical support and contraction force in the bronchial during breathing, and excessive ASM mass is related to airway hyper-responsiveness under asthmatic condition, which has been a treatment target (Zuyderduyn et al., 2008; Balestrini et al., 2021). As a chronical disease impacting a large population in the world, asthma can be characterized with airway inflammation, increased ASM mass and prolonged contraction in the bronchial (Zuyderduyn et al., 2008; Doeing and Solway, 2013). ERK also shows crucial roles in regulating ASM cell proliferation and interleukin expression in lymphocytes in asthma-related conditions (Burgess et al., 2008; Li et al., 2010). Here we applied fluorescence resonance energy transfer (FRET) biosensor to directly visualize cellular ERK activity induced by cyclic stretch in ASM cells which are under physiological stretch during breathing. The FRET biosensor allows to visualize the dynamic ERK activity in live cells along with subcellular resolution (Komatsu et al., 2011; Ponsioen et al., 2021), which provided the fine measurement of ERK activation during the cyclic stretch stimulation. Our work demonstrated in ASM cells that Ca^2+^ channels via actin cytoskeleton, particularly those channels on ER membrane, acted as the mechanosensing pathway to the downstream activation of ERK. Interestingly, the ER IP_3_R-dependent ERK activation by cyclic stretch was independent of upstream PLC-IP_3_ signal, indicating possibly more mechanical mechanism for the IP_3_R activation.

## Materials and Methods

### Chemical Reagents

2-Amino-ethoxydiphenyl borate (2-APB, 100 µM), Nifedipine (10 µM), LaCl_3_ (100 µM), Cytochalasin D (1 µM), Blebbistatin (20 µM), Nocodazole (1 µM), ML-7 (20 µM), the ROCK inhibitor Y27632 (20 µM), and phalloidin-TRITC were purchased from Sigma-Aldrich. Thapsigargin (TG, 10 µM) was purchased from Abcam, U73122 (10 µM) from MedChemExpress, and fibronectin from Corning. ITGB1 (Beta1) siRNA (Integrin siRNA, 30 nM) was purchased from Thermo Fisher Scientific.

### Cell Culture

Primary ASM cells were isolated from the tracheas of 6-8-week-old Sprague Dawley rats as described previously (approved by the Ethics Committee of Changzhou University on Studies Ethics, Grant No. NSFC 11532003) (Wang et al., 2016). The cells were maintained in low-glucose Dulbecco’s modified Eagle’s medium (DMEM, Sigma-Aldrich) supplemented with 10% fetal bovine serum (FBS, Thermo), 100 μg/ml penicillin, and 100 unit/ml streptomycin at 37°C with 5% CO_2_ in a humidified incubator.

### Construction of Nuclear Localized ERK FRET Biosensor (Nuc-ERK FRET)

To make nuclear localized ERK biosensor, the nuclear-exporting signal (NES) peptide in the construct was replaced with double nuclear-localized signal (NLS) peptide (2x Pro-Lys-Lys-Lys-Arg-Lys-Val). Briefly, ECFP DNA fragment with 2xNLS sequence at the C-terminal was amplified by PCR. Both the original construct and amplified ECFP fragment were digested by NotI and SalI restriction enzymes, followed by ligation of the two digested products together to generate Nuc-ERK FRET construct.

### Plasmid and siRNA Transfections

DNA plasmid and siRNA were transfected into ASM cells by using Lipofectamine3000 according to the manual protocol (Invitrogen). Briefly, cells were passaged in medium without antibiotics into 6-well plates the day before transfection. By following the protocol, 2.5 µg biosensor DNA, 4 µl P3000, and 4 µl Lipofectamine3000 were mixed to assemble Lipid-DNA particles for each well before adding it into the cell culture. The medium was changed in 8 h and cell imaging experiments were performed 40–64 h later.

For siRNA co-transfection, when cells reached 60%–80% confluency, siRNA (30 nM) was transfected into the cells with 4 µl Lipofectamine3000 reagent. The medium was changed in 8 h, and after siRNA transfection for 24 h, the ERK FRET biosensor DNA was further transfected into the cells.

### Verification of ITGB1 siRNA Transfection Efficiency by qPCR

The efficiency of ITGB1 siRNA transfection was assessed with mRNA expression using Real-Time Quantitative PCR (qPCR) assay. Total RNA from cultured ASM cells was extracted using the TRI Reagent RNA Isolation Reagent (#T9424, Sigma). Total RNA weighing 500 ng was applied to generate 1st strand cDNA by using the Revert Aid First Strand cDNA Synthesis Kit (#K1622, Thermo, MA). The sequences of associated qPCR primers for rat ITGB1 were derived from the previous report including GAA​TGG​AGT​GAA​TGG​GAC​AGG​AG (ITGB1 forward), CAG​ATG​AAC​TGA​AGG​ACC​ACC​TC (ITGB1 reverse), and the control GAPDH primers AGG​TCG​GTG​TGA​ACG​GAT​TTG (forward) and GGG​GTC​GTT​GAT​GGC​AAC​A (reverse) (Luo et al., 2018). The primers were synthesized from General Biosystems (Anhui, China), and PowerUp SYBR Green Master Mix (#A25742, Applied Biosystems, CA) was used for PCR amplification. The reaction was run in the qRT-PCR system (StepOnePlus, Applied Biosystems) with 1 µL of the cDNA in a 10 µL reaction according to the manufacturer’s instructions. Calibration and normalization were done using the 2^−∆∆CT^ method, where ∆∆CT = CT (target gene) -CT (reference gene), and CT referred to the PCR cycle number by reaching the defined fluorescence intensity. Fold changes in mRNA expression were calculated based on the resulting CT values from three independent experiments.

### Mechanical Stretch

After DNA transfection for 24 h, cells were detached using Accutase solution (Sigma), and transferred to collagen I and fibronectin (40 μg/ml) double-coated 6-well BioFlex plates (Flexcell International Corporation, Hillsborough, NC, United States). After which the cells were cultured in the medium containing 1% FBS for 12–16 h and reached 60–80% confluence before the mechanical stretch. Cyclic stretch was applied with a 0.5-Hz sinusoidal curve at 12% elongation by using a Flexcell^®^ FX-5000^TM^ Tension System (Flexcell^®^ International Corporation). The computer program-controlled bioreactor used vacuum and positive air pressure to apply cyclic strain to cells cultured on flexible-bottomed BioFlex plates. After the durations of cyclic stretch, the BioFlex plates with cells were moved from the Flexcell incubator to Zeiss microscopy for FRET imaging. The images of a group of cells were acquired within 10 min for each well.

### FRET Microscopy Imaging

The processes of FRET imaging and quantification were similar to our recent descriptions (Ouyang et al., 2019; Yao et al., 2020). Briefly, the Zeiss microscopy system (Zeiss Cell Observer) was equipped with the functions of multi-positions, fine auto-focusing, and automatic-switchable dichroic rotator. The scope stage was supplemented with an incubator box (Zeiss) to maintain temperature at 37°C and 5% CO_2_ for live cell samples. During FRET image acquisitions through ECFP and FRET (YPet) channels, the parameters for excitation filter and dichroic mirror were 436 ± 10 and 455 nm, respectively, and the emission filters of ECFP and FRET (YPet) channels were 480 ± 20 nm and 535 ± 15 nm, respectively.

**FIGURE 1:**
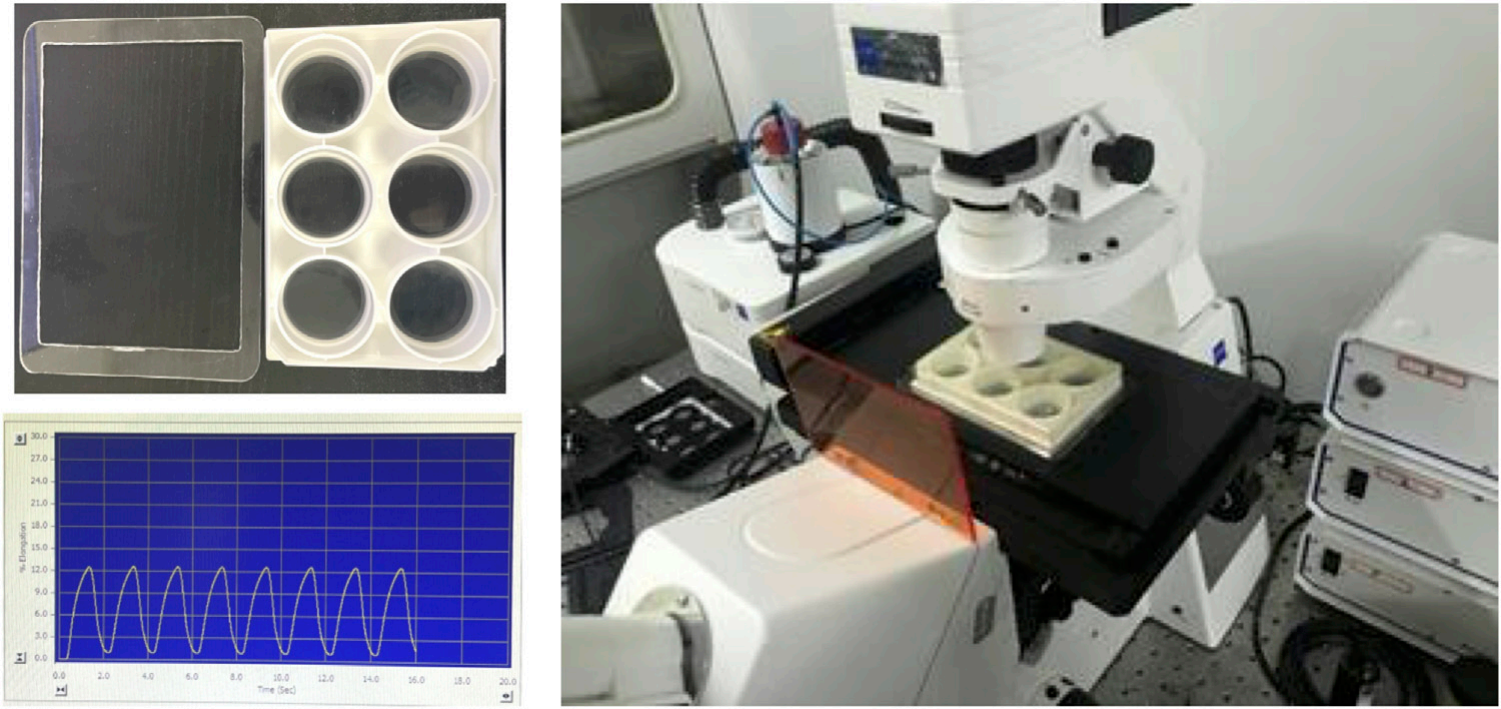
The setup to load the BioFlex plate on the microscope stage for FRET imaging, and the sample of 0.5-Hz sinusoidal curve from cyclic stretch by using the Flexcell^®^ System.

To take the FRET images before and after the cyclic stretch, we made an accessory holder with acrylic plate to load the BioFlex plate on the microscopy stage, as shown in Figure 1. Before starting the stretch, 15–20 positions of fluorescent cells were selected per well, and FRET images were acquired as the pre-stretch condition. After the duration (0.5–1 h) of cyclic stretch, the same positions of cells were reloaded with minor adjustments, and the FRET images were taken within 10 min for each well before the next cycle.

### FRET Quantification and Statistical Analysis

FRET quantifications were processed using the Wang Lab (UCSD)-developed software package FluoCell in MATLAB (available on http://github.com/lu6007/fluocell) (Qin et al., 2019). Fluorescence signals from ECFP and FRET (YPet) images were measured after background subtractions, and the ratio of the two channels was calibrated in the pixel-to-pixel manner. Data procession and statistical analysis were done by the software of Graphpad Prism 6, and Excel. The quantified FRET data from a group of cells was expressed in curves (Mean ± S.E.M.), and scattering dots (Mean ± S.D.). *, **, *** and **** indicate *p* < 0.05, 0.01, 0.001, and 0.0001 from Student’s t-test, which was applied for significant difference analysis. Multiple times of t-test analysis done between the control and one experimental group were carried for variable experimental conditions using Graphpad Prism 6. All described FRET experiments have been repeated independently on different days with similar conclusions, and statistical quantifications were performed based on the data acquired from different time.

## Results

### Cyclic Mechanical Stretch-Induced ERK Activation Measured by FRET Biosensor

**FIGURE 2:**
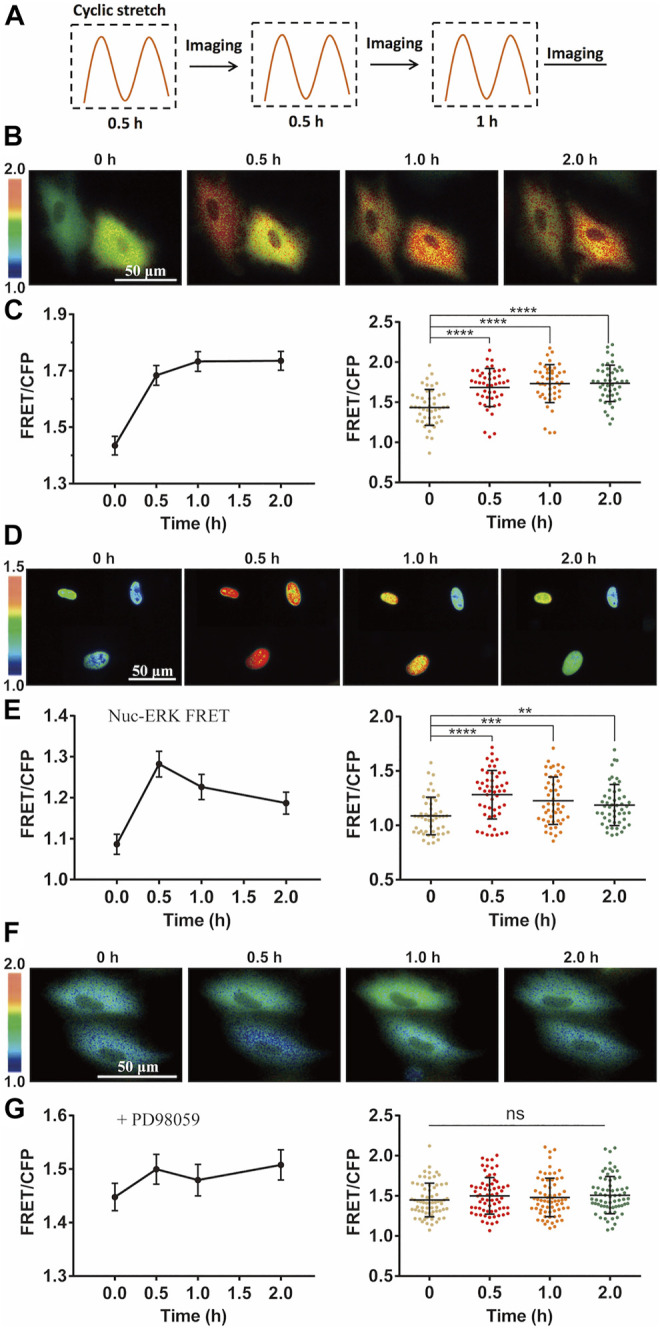
Cyclic stretch-induced ERK activation by FRET measurements. FRET/ECFP emission ratiometric images show the activation of ERK by cyclic stretch in ASM cells pre-incubated in 1% FBS culture medium. Scale bar = 50 µm. **(A)** Illustrated experimental procedures of cyclic stretch stimulation (with a 0.5-Hz sinusoidal curve at 12% elongation deformation) followed by FRET imaging in multiple cycles. **(B,C)** ERK FRET ratio (YPet/ECFP) images **(B)**, and quantifications of the FRET changes in curves (Mean ± S.E.M.) and scattering dots (Mean ± S.D. *n* = 46) **(C)** after cells were stimulated with cyclic stretch for the indicated time. **(D,E)** FRET images with Nuc-ERK FRET biosensor in cells **(D)**, and their statistical quantifications (*n* = 50) **(E)** after cyclic stretch at the indicated time points. **(F,G)** ERK FRET images **(E)** and FRET statistical quantifications (*n* = 67) **(G)** in cells pre-treated with 10 µM PD98059 inhibitor for 1 h. Student’s t-test was performed between the control and one experimental group, and multiple rounds of t-test were done for the variable experimental conditions. ∗, ∗∗, ∗∗∗, and ∗∗∗∗ indicate *p* < 0.05, 0.01, 0.001, and 0.0001 from Student’s t-test analysis while ‘ns’ for no significant difference, and so on through the paper.

To understand how mechanical stimulation from cyclic stretch activates biochemical signals in live cells, we applied FRET biosensor to measure ERK kinase activity in ASM cells. According to the previous study (Takahara et al., 2014), 12% strain deformation with a 0.5-Hz sinusoidal curve was within the physiological scale and was applied on the cells seeding on the elastic membrane by the Flexcell^®^ Tension System, which could induce alignment of cells perpendicularly to the stretch direction in 7.5 h (Supplementary Figure S1). In regarding the inapplicability of visualizing the FRET signals on the microscope simultaneously during the cyclic stretch, the procedures were carried out in the following process: the FRET images were taken after 0.5 h-stretch followed by another 0.5 h-stretch and imaging, and then continued with one more 1 h-stretch and imaging (depicted in Figure 2A). The imaging was usually done within 10–30 min between the three times of stretch, and images of similar cell positions were acquired each time.

Intracellular ERK in the cytoplasm was activated by the mechanical stretch, as seen by the FRET changes (Figure 2B). FRET quantification from a group of cells showed apparent activity increase with statistical significance (Figure 2C). As control, no apparent FRET change was observed in the similar imaging procedure without stretch stimulation (Supplementary Figure S2). The platelet-derived growth factor (PDGF) regulates ERK activity in ASM cell proliferation and also inflammation in asthmatic condition (Lee et al., 2001; Kardas et al., 2020). The magnitude of ERK FRET change (∼15–20%) from the mechanical stretch was comparable to PDGF-stimulated FRET change in ASM cells (Supplementary Figure S3). In consideration of ERK shuttling between the cell cytoplasm and nucleus, we modified the ERK biosensor to generate nuclear localized version Nuc-ERK FRET. As shown in Figures 2D,E, nuclear ERK was also activated by the mechanical stretch, which indicates the cytoplasmic increase is not due to nuclear ERK transportation. Hence, we used cytoplasmic FRET measurement to represent the whole cells in the following study. The ERK activation reported by FRET signals was verified by increased phosphorylation level of ERK with phospho-ERK antibody immunoblotting under the cyclic stretch (Supplementary Figure S4). The 12% strain deformation of ASM cells is within the physiological range of stretch magnitude (Takahara et al., 2014), which is also seen by the maintained cell shape (Figure 2B) and actin stress fibers (Supplementary Figure S5) after stretch. We further pretreated the cells with ERK inhibitor PD98059, which abolished stretch-induced FRET signals (Figures 2F,G), hence the FRET changes truly reported ERK activity.

### Ca^2+^ Channel-Dependent ERK Activation by Cyclic Stretch

**FIGURE 3:**
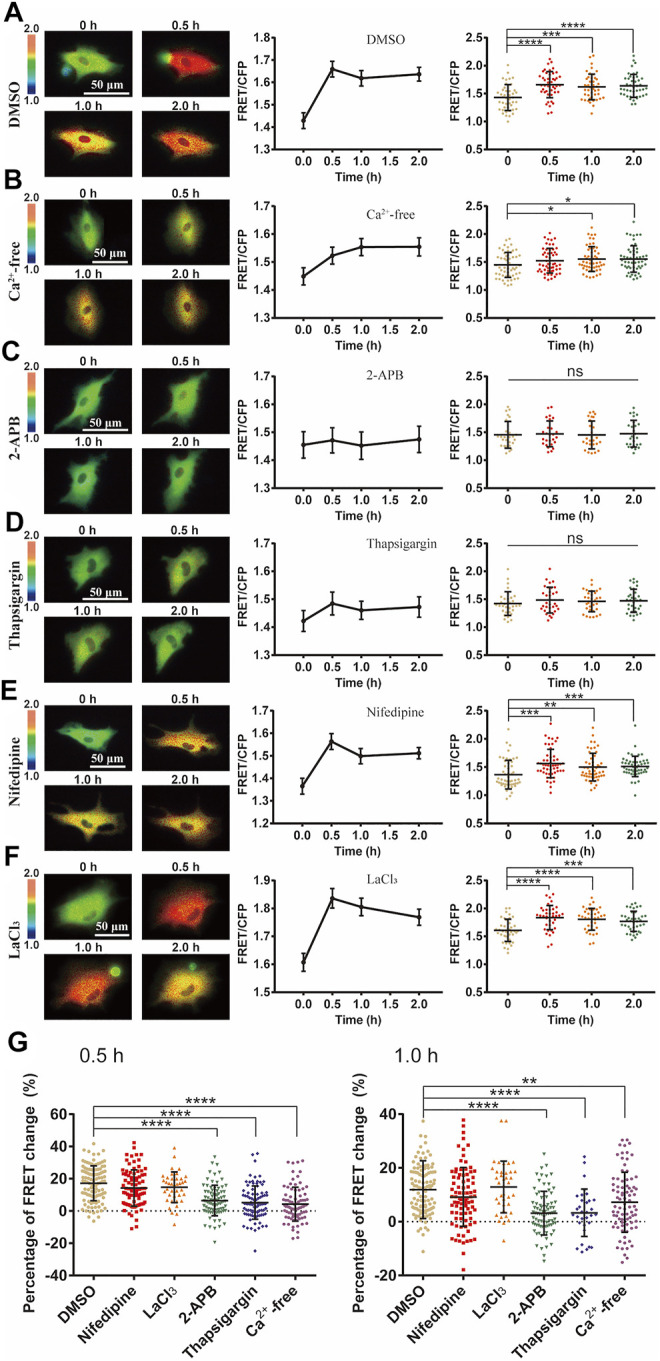
Ca^2+^ channel-dependent ERK activation by cyclic stretch. ASM cells were pretreated with inhibitors of variable Ca^2+^ channels followed by cyclic stretch and imaging. FRET ratiometric images along with FRET change quantifications are shown for each condition before and after cyclic stretch. **(A–F)** FRET changes of ERK biosensor in cells under control (0.1% (v/v) DMSO) condition (N = 42) **(A)**; in Ca^2+^-free culture medium (*n* = 53) **(B)**; pre-treated with 100 μM 2-APB, an IP_3_R blocker (*n* = 26) **(C)**; 10 μM Thapsigargin, a SERCA pump blocker (*n* = 32) **(D)**; 10 μM Nifedipine, a L-type Ca^2+^ channel blocker (*n* = 52) **(E)**; 100 μM LaCl_3_, store-operated Ca^2+^ channel (SOC) blocker (*n* = 39) **(F)**. **(G)** Statistical quantifications for the percentage changes of FRET ratio (YPet/ECFP) in ASM cells before and after 0.5 h cyclic (left graph) and total 1 h stretch (right graph). The conditions from **(A–F)** were pre-treated with DMSO (control), Nifedipine, LaCl_3_, 2-APB, Thapsigargin or in calcium-free medium, respectively. *n* = 108, 87, 39, 84, 90/32, 83 in the indicated order. Student’s t-test was performed between the control and one experimental group, and multiple rounds of t-test were done for the variable experimental conditions.

We then investigated the mechanosensitive components in transducing the mechanical stimulation into biochemical signals in cells. Ca^2+^ channels located on plasma membrane or endoplasmic reticulum (ER) are sensitive to mechanical stimulations (Kim et al., 2015). By switching the culture medium to Ca^2+^-free one before cyclic stretch, ERK activation was inhibited with a delayed response (Figures 3A,B). This suggests that Ca^2+^ channels on the plasma membrane had significant contributive role while not essential to the mechanical activation of ERK. However, by selective chemical inhibition of IP_3_R Ca^2+^ channel with 2-APB, or SERCA pump with Thapsigargin on ER membrane (Wang et al., 2021), the mechanical activation of ERK was almost blocked (Figures 3C,D), indicating that Ca^2+^ release from the ER store had an essential role. In contrast, there was little effect on the ERK activation by inhibition of L-type Ca^2+^ channel with nifedipine, or store-operated Ca^2+^ channel (SOC) with LaCl_3_ on plasma membrane (Parekh and Putney, 2005; Wang et al., 2021) (Figures 3E,F). From statistical comparison of the FRET changes (in percentage) after 0.5 h stretch and total 1 h stretch (Figure 3G), inhibition of IP_3_R or SERCA pump on ER membrane had the most significant impact on the mechanical activation of ERK, followed by inhibiting extracellular Ca^2+^ uptakes with reduced delayed response, while L-type Ca^2+^ channel and SOC on the plasma membrane were not actively involved into the ERK activation.

### Cyclic Stretch-Induced ERK Activation Not Dependent on PLC-IP_3_ Signal

**FIGURE 4:**
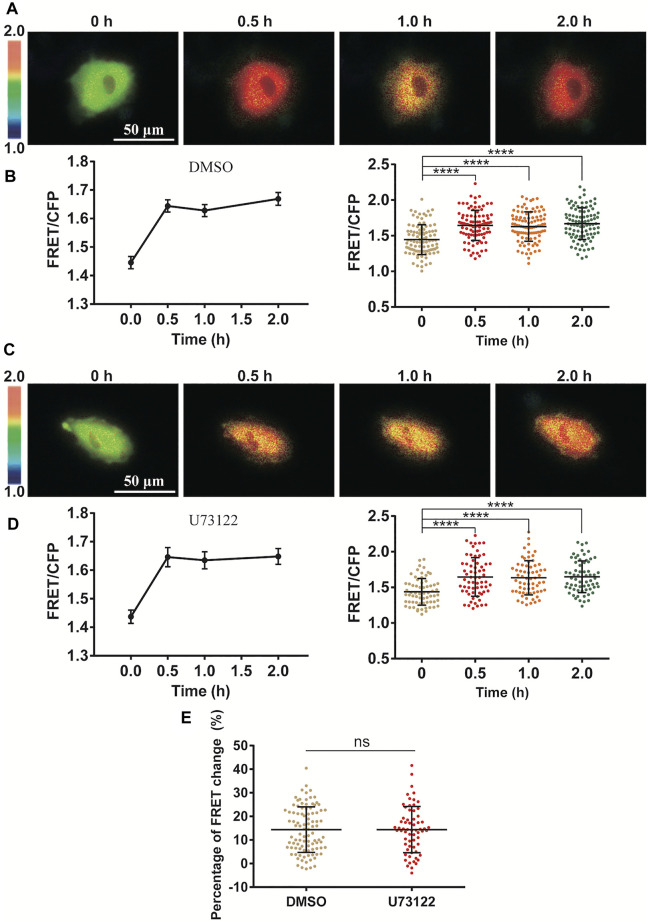
Independence of PLC-IP_3_ signal for cyclic stretch-induced ERK activation. ASM cells were pretreated with PLC inhibitor U73122 (10 µM) for 1.5 h followed by cyclic stretch. **(A–D)** FRET ratiometric images and quantification of FRET changes before and after cyclic stretch under the conditions of control (DMSO) **(A,B)** and U73122 treatment **(C,D)**. **(E)** Statistical comparison of FRET changes between the control (*n* = 96) and U73122 (*n* = 65) treatment after 0.5 h cyclic stretch, which is based on FRET data from three independent experiments. Student’s t-test was performed between the control and one experimental group.

Since Ca^2+^ release through IP_3_R channel on ER membrane was essential in the stretch activation of ERK (Figure 3), we further looked at whether inositol 1,4,5-triphosphate (IP_3_) production was necessary in turning on the IP_3_R channel. As a classic pathway, phospholipase C (PLC) is an enzyme that hydrolyzes PIP2 on the plasma membrane to generate IP_3_, which results in opening the IP_3_R Ca^2+^ channel (Bartlett et al., 2020). In our experiments, inhibition of PLC activity with specific inhibitor U73122 had little effect on the cyclic stretch-induced ERK activation in comparison to the control group (Figures 4A–E). This indicates that the PLC-IP_3_ signaling pathway was not essential in mechanical stimulation of IP_3_R Ca^2+^ channel for ERK activation.

### The Regulatory Role of Actin Cytoskeleton on the Mechanical Activation of ERK

**FIGURE 5:**
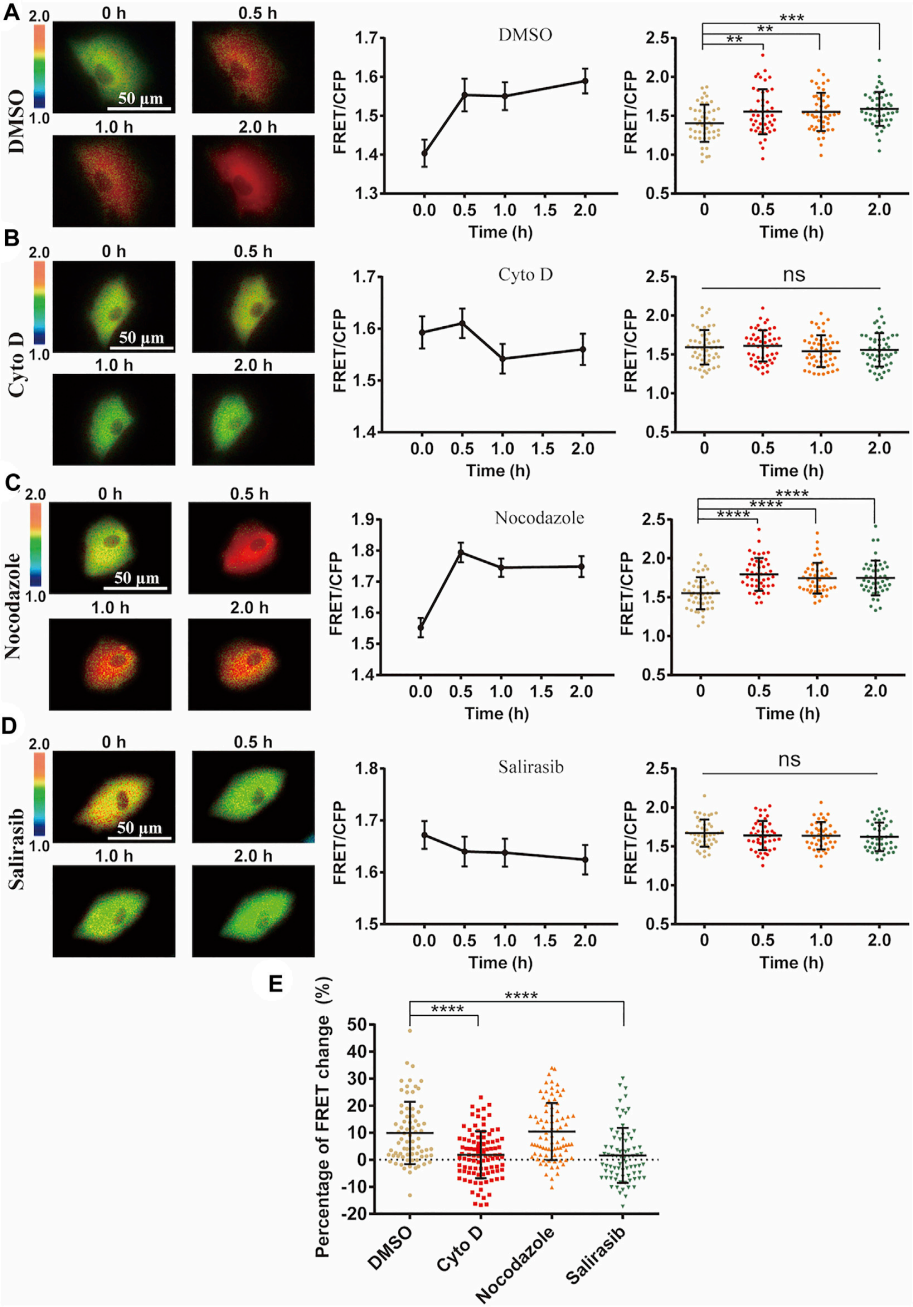
Role of cell cytoskeleton on cyclic stretch-induced ERK activation. ASM cells were pretreated with cytoskeleton or Ras inhibitors, followed by cyclic stretch and imaging. FRET ratiometric images along with change quantifications are shown for each condition before and after cyclic stretch. **(A–D)** ERK FRET ratiometric images and statistical quantifications of FRET changes in ASM cells pretreated with DMSO (*n* = 47) **(A)**, 1 μM Cyto D (N = 51) **(B)**, 1 μM Nocodazole (*n* = 45) **(C)**, and 75 µM Ras inhibitor Salirasib (*n* = 43) **(D)**. **(E)** Statistical comparisons of FRET percentage changes in ASM cells pre-treated with DMSO, Cyto D, Nocodazole, and Salirasib (*n* = 73, 99, 82, 77) **(A–D)** before and after 0.5 h cyclic stretch, based on image data from two independent experiments. Student’s t-test was performed between the control and one experimental group.

Actin cytoskeleton often serves for mechanical transmission within cells (Wei et al., 2020). We hence checked the role of cytoskeleton in the mechanical activation of ERK by FRET measurements. After loss of actin cytoskeleton integrity with Cytochalasin D (Cyto D) treatment which inhibited actin filament polymerization (Supplementary Figure S5), cyclic stretch-induced ERK activation was almost completely inhibited (Figures 5A,B). Contrarily, inhibition of microtubule cytoskeleton with Nocodazole treatment did not show much effect (Figure 5C). The small GTPase Ras acts upstream of ERK through Ras-Raf-MEK-ERK signaling (Rudzka et al., 2021). The stretch-induced ERK activation was suppressed by pretreatment with Ras inhibitor Salirasib (Figure 5D), supporting that mechanically induced Ca^2+^ signal activated ERK through Ras pathway.

### Cyclic Stretch-Induced ERK Activation Independent of Integrin β1

**FIGURE 6:**
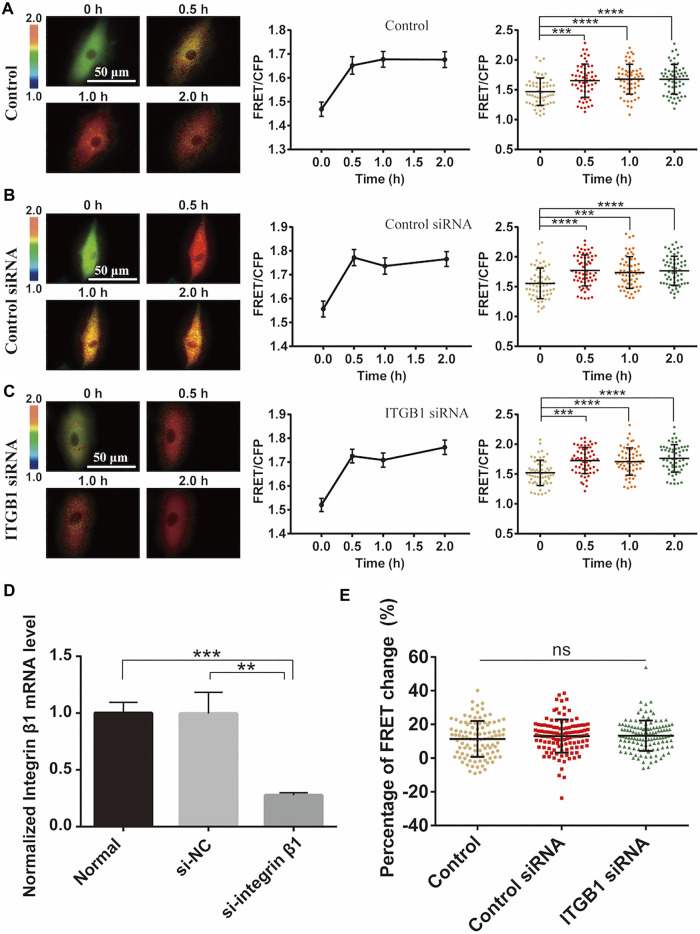
Integrin β1 (ITGB1) signal in cyclic stretch-induced ERK activation. ASM cells were transfected with ITGB1 inhibitory siRNA, followed by cyclic stretch and imaging. **(A–C)** FRET ratiometric images along with FRET change quantifications are shown before and after cyclic stretch in ASM cells without transfection **(A)**, or transfected with control scramble siRNA **(B)** and ITGB1 siRNA **(C)** (*n* = 58, 60, 58), respectively. **(D)** qPCR quantification from three experimental data confirmed the reduced level of ITGB1 mRNA expression after siRNA transfection. **(E)** Statistical comparisons of FRET percentage changes in cells without transfection, or transfected with control or ITGB1 siRNA (*n* = 103, 116, 121) **(E)** before and after 0.5 h stretch. Student’s t-test was performed between the control and one experimental group.

Integrins are mechanosensitive molecules on plasma membrane (Sun et al., 2016; Potla et al., 2020). Integrin β1 (ITGB1) is a predominant subunit in forming functional heterodimers with integrin α1, α2…α11, and α_v_ subunits (Cai et al., 2021), so we tried to regulate the representative β1 to check its role in the cyclic stretch-induced activation of ERK. After knockdown of ITGB1 expression with siRNA in cells, there was no obvious impact on the cyclic stretch-induced ERK activation in comparison to the control groups (Figures 6A–C). The reduced mRNA expression of ITGB1 was confirmed by qPCR measurements (Figure 6D). Statistical quantifications confirmed no significant change in the mechanical stretch-activated ERK by ITGB1 knockdown (Figure 6E). These data indicate that integrin α_(x)_β1 was not the primary mechanosensor in the mechanical activation of ERK.

## Discussion

The physical stretches seem critical for body health and organ homeostasis, whereas extreme stretch can be hurting as seen in sports and clinical applications (Crawford et al., 2021; Kaczka, 2021). By applying the Flexcell^®^ tension system, we investigated cyclic stretch-induced ERK activation in ASM cells, particularly paying attention to the mechanosensitive pathway for the mechanical stimulation to biochemical signal transduction.

FRET measurements showed that ERK was efficiently activated by the mechanical stretch (Figures 2A–F). Previous studies have shown that Ca^2+^ signals in stem cells are sensitive to mechanical stimulations (Horie et al., 2016), so we checked whether Ca^2+^ channels are among the mechanosensing components for the ERK activation. Inhibition of extracellular Ca^2+^ uptakes partially reduced the mechanical activation of ERK while blocked Ca^2+^ release from the ER store had complete inhibition (Figures 3A–D). L-type Ca^2+^ channel or SOC channel on the plasma membrane was not actively engaged into the induced ERK activation (Figures 3E,F). Therefore, the mechanical stretch-induced activation of Ca^2+^ channels was essential for downstream ERK activation, which is within the mechanosensing pathway.

The Ca^2+^ channels on the ER membrane are essential for the mechanical activation of ERK (Figures 3C,D), and this observation is consistent with the previous report that Ca^2+^ signal from the ER store is induced by pulling force on the cell surface by optical laser tweezers (Kim et al., 2015). In considering that inhibition of IP_3_R channel on ER blocked the ERK activation (Figure 3C), and IP_3_ is the ligand to turn on IP_3_R channel, however, inhibition of PLC-IP_3_ pathway had little effect (Figures 4A–E). Therefore, PLC-dependent IP_3_ production was not essential in the ERK activation, indicating possibly more mechanical mechanism for ER IP_3_R channel activation from cyclic stretch stimulation.

Actin cytoskeleton can pass the stretch force within the cells. Loss of actin cytoskeleton resulted in abolishing the mechanical activation of ERK, but not by loss of microtubule cytoskeleton (Figure 5), indicating that actin cytoskeleton has the crucial role on ERK activation. Possibly actin cytoskeleton passed the force to trigger the ER Ca^2+^ signals (Kim et al., 2015), which is also suggested from our data (Figures 5A–E). Ca^2+^ signals can further activate PKC to induce Ras-Raf-MEK-ERK pathway (Liao et al., 2020; Yan et al., 2020), and our data showed that inhibition of Ras truly blocked the mechanical activation of ERK (Figures 5D,E). These data indicate that actin cytoskeleton belongs to the mechanosensing component for ERK activation.

Integrins are mechanosensitive molecules and regulate focal adhesions to transmit the force inside cells (Michael et al., 2009). Integrin subunit β1 forms functional heterodimers with variable integrin subunits α_(x)_, so we regulated β1 to check its role in the stretch-induced ERK activation. Interestingly, by reduction of β1 expression with siRNA, there was no apparent change in the ERK activation level (Figure 6). Since cells were attached well under the siRNA transfection, the stretch force may be still transmitted efficiently to activate ERK. Previous studies reported that mechanical stretch up-regulates integrin β_1D_ expression to result in FAK and RhoA activations (Zhang et al., 2007), while ERK activation by mechanical stretch is independent of FAK (Hsu et al., 2010). Hence, our observation seems consistent with that. Therefore, integrin α_(x)_β1 did not act essentially as the primary mechanosensing component in this stretch activation of ERK in ASM cells.

## Conclusion

**FIGURE 7:**
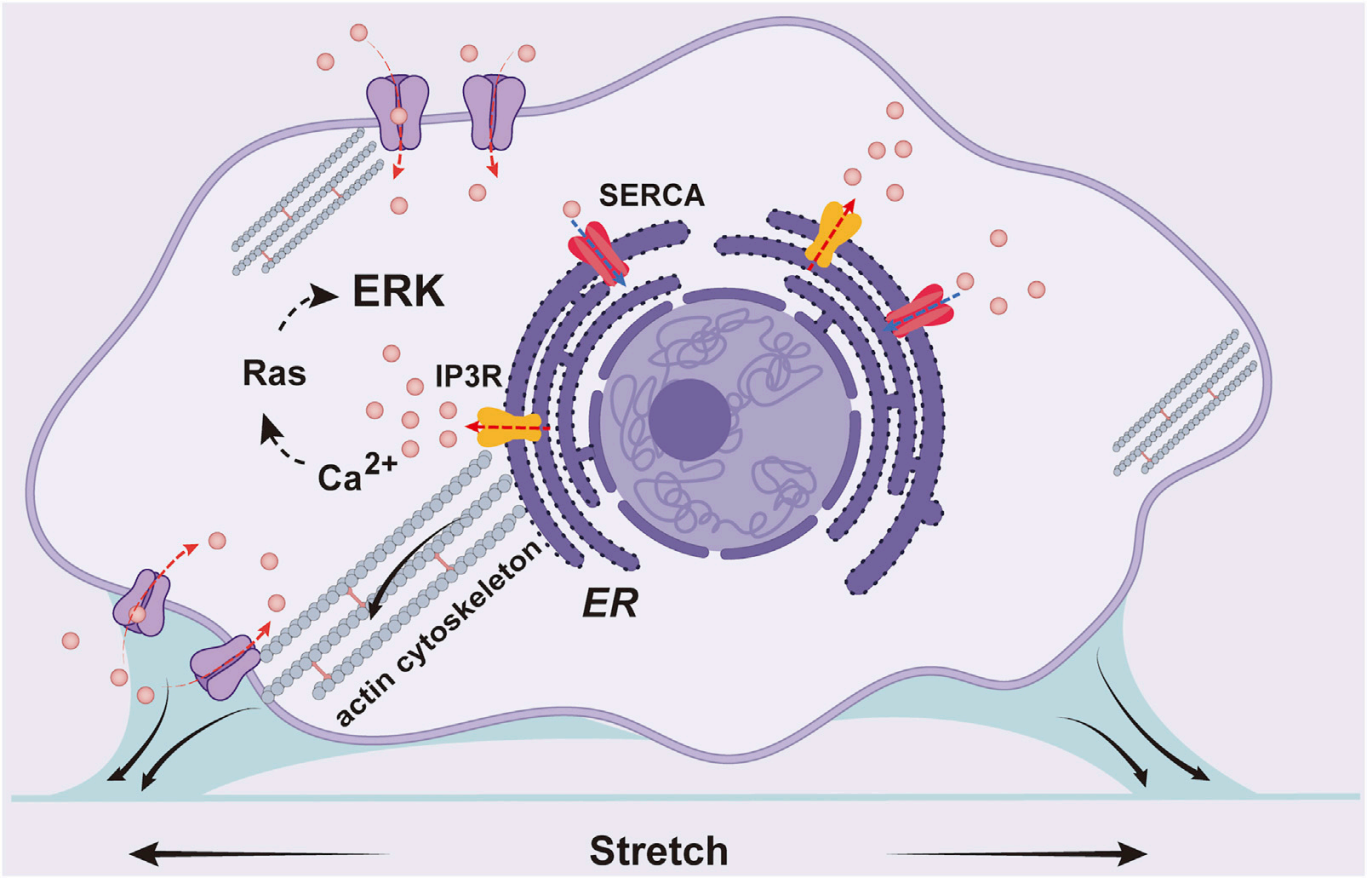
A schematic illustration for the mechanical activation of ERK in ASM cells. The cyclic stretch applied on the cells activates Ca^2+^ channels on the plasma and ER membranes including IP_3_R and SERCA pump, likely mediated by force transmission through actin cytoskeleton, and Ca^2+^ signals further trigger downstream pathway to activate ERK.

In summary, we visualized cyclic stretch-induced ERK activation by FRET biosensor in ASM cells, and provided molecular insights for the mechanosensitive pathway from mechanical stimulation to ERK biochemical activity. Data shows that Ca^2+^ channels and actin cytoskeleton are essential mechanosensing components, while integrin β1 is not essential in the mechanotransduction. Together with previous work, our experimental results support the following hypothesis for the mechanical activation of ERK in ASM cells (Figure 7): the cyclic stretch applied on the cells activates Ca^2+^ channels mechanically on the plasma and ER membranes, likely mediated by force transmission through actin cytoskeleton, and Ca^2+^ signals further trigger downstream signaling pathways including ERK activity in the cells.

The contributions of this work to the cyclic stretch study may be described as follows: 1) methodologically, directly visualized ERK activation by cyclic stretch in live cells at different time points with FRET biosensor, whereas previous studies were mostly done in cell lysis by using antibody detections; 2) identified that calcium channels, particularly IP_3_R channel and SERCA pump on ER membrane, but not integrin α_(x)_β1 signals, are the primary mechanosensitive components for ERK activation, which is likely mediated *via* force transmission through actin cytoskeleton; 3) the ER IP_3_R channel-dependent ERK activation does not rely on the classic upstream phospholipase C-IP_3_ signal, indicating possibly a more mechanical mechanism for IP_3_R activation by cyclic stretch. Therefore, this work provides progress in understanding this mechanical-biomechanical coupling process.

## Data Availability

The original contributions presented in the study are included in the article/Supplementary Material, further inquiries can be directed to the corresponding authors.
