# Supplementary material for: FRET Visualization of Cyclic Stretch-Activated ERK via Calcium Channels Mechanosensation While Not Integrin β1 in Airway Smooth Muscle Cells
Source: Front Cell Dev Biol. 2022 May 19;10:847852. doi: 10.3389/fcell.2022.847852 (PMC9162487; doi:10.3389/fcell.2022.847852)
Supplement: Supplementary file 1 [file DataSheet1.pdf]

Supporting information

FRET visualization of cyclic stretch-activated ERK via calcium channels mechanosensation while not integrin β1

Xin Fang[1], Kai Ni[1], Jia Guo[1], Yaqin Li[1], Ying Zhou[1], Hui Sheng[1], Bing Bu[1], Mingzhi Luo[1], Mingxing Ouyang[1,*], Linhong Deng[1,*]

[1]Institute of Biomedical Engineering and Health Sciences, School of Pharmacy & School of Medicine, Changzhou University, Changzhou City, China

The Supporting Information includes 5 supporting figures.

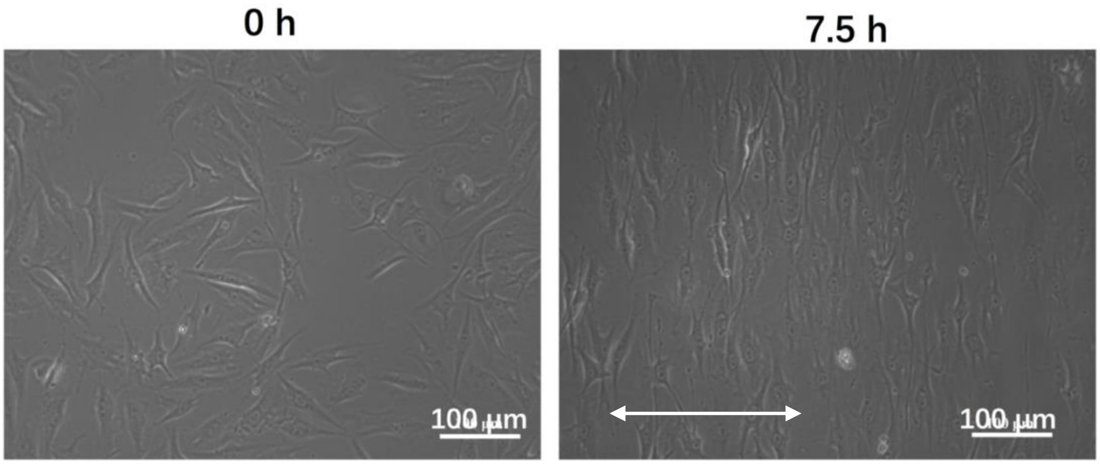

Figure S1. Cyclic stretch induced alignment of cultured ASM cells perpendicularly to the direction of stretch force. Cell images were taken before and after 7.5 h stretch around the periphery areas of the BioFlex plate wells. Cells show perpendicular alignment to the stretch direction (indicated by the arrow).

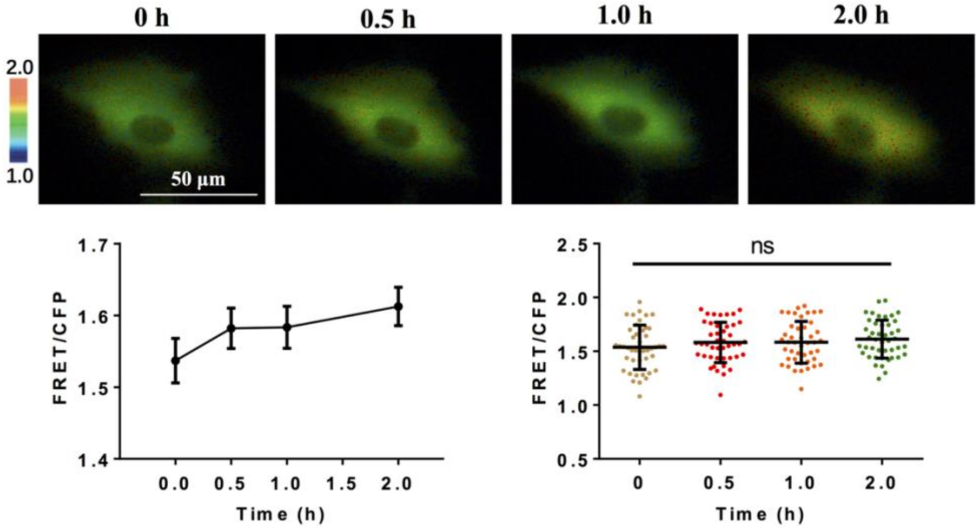

Figure S2. Undetectable FRET change of ERK biosensor in ASM cells without the cyclic stretch. As control experiment, ASM cells expressing ERK FRET biosensor were placed into the FlexCell system without stretch, followed with the similar procedures of imaging. Representative FRET ratiometric images were shown along with statistical quantifications from the cell group.

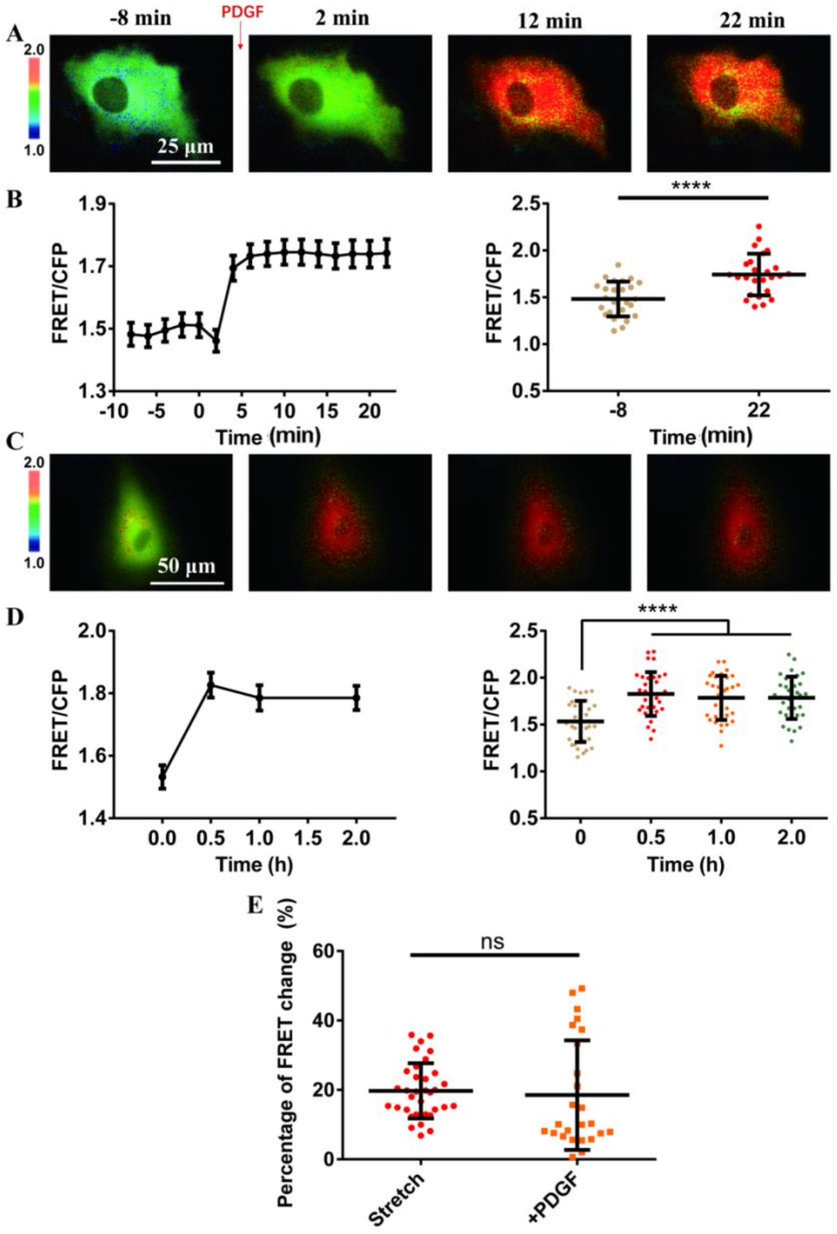

Figure S3. Comparable magnitudes of ERK activation by cyclic stretch and growth factor PDGF stimulations. For magnitude comparison of ERK activations, ASM cells were stimulated with chemical PDGF (50 ng/ml) or cyclic stretch, followed with FRET quantifications. **(A-D)** PDGF-induced FRET change (**A, B**) and cyclic stretch-induced FRET change (**C, D**) in ASM cells expressing ERK biosensor. **(E)** Statistical comparison of ERK FRET changes between cyclic stretch and PDGF stimulations within 0.5 h.

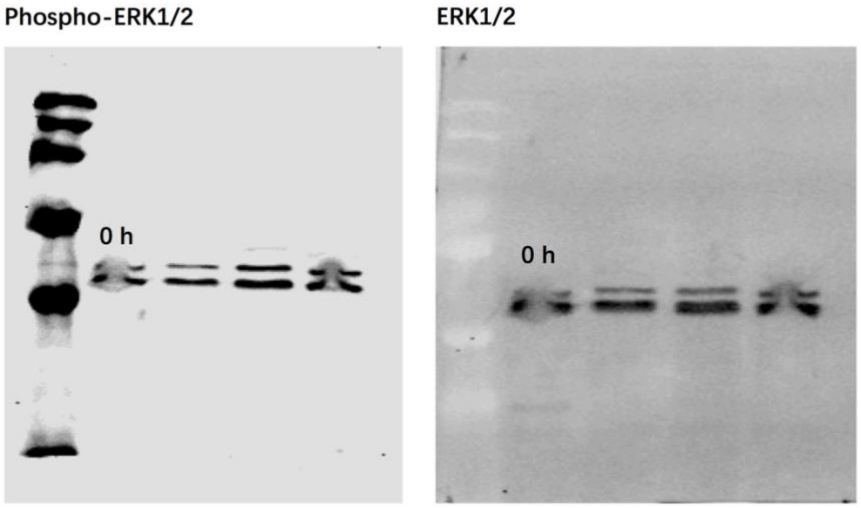

Figure S4. Cyclic stretch-induced ERK activation detected by phospho-ERK antibody. After cyclic stretch at the durations of 0, 0.5, 1.0, 2.0 h, the total proteins from ASM cell lysates were resolved by SDS-PAGE (sodium dodecyl sulfate polyacrylamide gel electrophoresis), and then transferred to the nitrocellulose membrane, followed by sequential staining with phospho-ERK1/2 antibody (left panel) and total ERK1/2 antibody (right panel).

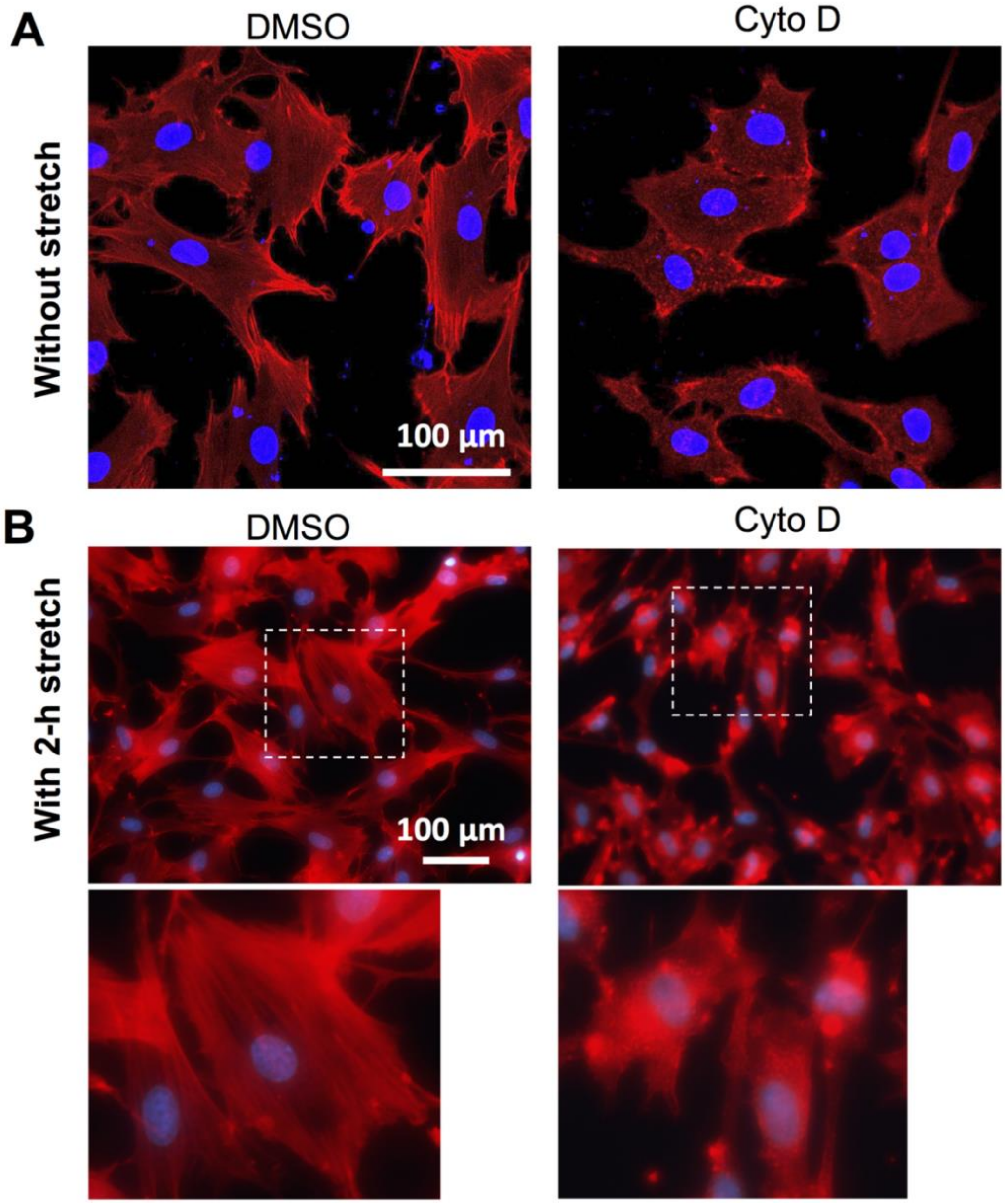

Figure S5. Actin fiber staining with phalloidin-TRITC. (A, B) ASM cells were treated with DMSO (control), or Cyto D (1 µM) for 2 h, followed without (**A**) or with (**B**) another 2-h cyclic stretch before phalloidin-TRITIC (actin fibers) and DAPI (nuclei) double staining. Images were taken by confocal microscopy with 100x oil objective on glass (**A**), or by epifluorescence microscopy with 40x objective on BioFlex plate (inaccessible by the oil objective) (**B**). Some enlarged local regions show visible or invisible actin fibers in (**B**).
